# Western diet is associated with a smaller hippocampus: a longitudinal investigation

**DOI:** 10.1186/s12916-015-0461-x

**Published:** 2015-09-08

**Authors:** Felice N. Jacka, Nicolas Cherbuin, Kaarin J. Anstey, Perminder Sachdev, Peter Butterworth

**Affiliations:** Division of Nutritional Psychiatry Research, IMPACT Strategic Research Centre, Deakin University, Geelong, Australia; Department of Psychiatry, The University of Melbourne, Melbourne, Australia; Centre for Adolescent Health, Murdoch Children’s Research Institute, Melbourne, Australia; Black Dog Institute, Sydney, Australia; Centre for Research on Ageing, Health and Wellbeing, Research School of Population Health, The Australian National University, Canberra, Australia; Centre for Healthy Brain Ageing (CHeBA), School of Psychiatry, Faculty of Medicine, University of New South Wales, Sydney, Australia

**Keywords:** Brain derived neurotrophic factor, Hippocampus, Diet, Magnetic resonance imaging, Neurogenesis, Nutrition

## Abstract

**Background:**

Recent meta-analyses confirm a relationship between diet quality and both depression and cognitive health in adults. While the biological pathways that underpin these relationships are likely multitudinous, extensive evidence from animal studies points to the involvement of the hippocampus. The aim of this study was to examine the association between dietary patterns and hippocampal volume in humans, and to assess whether diet was associated with differential rates of hippocampal atrophy over time.

**Methods:**

Data were drawn from the Personality and Total Health Through Life Study and focused on a subsample of the cohort (n = 255) who were aged 60–64 years at baseline in 2001, completed a food frequency questionnaire, and underwent two magnetic resonance imaging scans approximately 4 years apart. Longitudinal generalized estimating equation linear regression models were used to assess the association between dietary factors and left and right hippocampal volumes over time.

**Results:**

Every one standard deviation increase in healthy “prudent” dietary pattern was associated with a 45.7 mm^3^ (standard error 22.9 mm^3^) larger left hippocampal volume, while higher consumption of an unhealthy “Western” dietary pattern was (independently) associated with a 52.6 mm^3^ (SE 26.6 mm^3^) smaller left hippocampal volume. These relationships were independent of covariates including age, gender, education, labour-force status, depressive symptoms and medication, physical activity, smoking, hypertension and diabetes. While hippocampal volume declined over time, there was no evidence that dietary patterns influenced this decline. No relationships were observed between dietary patterns and right hippocampal volume.

**Conclusions:**

Lower intakes of nutrient-dense foods and higher intakes of unhealthy foods are each independently associated with smaller left hippocampal volume. To our knowledge, this is the first human study to demonstrate associations between diet and hippocampal volume concordant with data previously observed in animal models.

## Background

Unhealthy dietary habits are recognised as major contributors to many of the common noncommunicable diseases, including cardiovascular disease, cancer and diabetes [1, 2]. There has been a global shift toward increased intake of fast foods and sugar-sweetened beverages [3], and this increasingly “obesogenic” environment explains much of the development of the well-documented obesity epidemic [2].

Emerging evidence also supports a role for poor diet in promoting mental disorders, including depression and dementia [4, 5] and there are now extensive data showing that a healthy diet is inversely related to the risk for both depression and cognitive decline [6, 7]. We have previously shown that dietary patterns characterized by higher intakes of nutrient-dense foods, such as vegetables, fruit, whole grains and fish, are associated with a reduced prevalence and risk for depressive symptoms and disorders, whereas dietary patterns higher in saturated fats and refined carbohydrates—a Western-style dietary pattern—are independently associated with increased depression and depressive symptoms, both cross-sectionally and over time [8–10]. While the biological pathways that underpin these relationships are likely multitudinous, and include inflammation [11], oxidative stress [12] and the gut microbiome [13], extensive evidence from animal studies points to the importance of the hippocampus in the association between diet and mental and cognitive health.

The hippocampus is a brain structure associated with both learning and memory, as well as mood regulation, and is specifically implicated in depression [14]. It is also one of only two areas of the brain where adult neurogenesis is prevalent. Environmental factors such as exercise, enriched environments and caloric restriction have been shown to increase adult hippocampal neurogenesis, while stress, low grade inflammation [15], oxidative stress and ageing appear to decrease it [16]. Hippocampal volume is reduced in adults with depression [17], while antidepressants appear to increase neurogenesis [18] and hippocampal volumes [19]. Moreover, hippocampal neurogenesis appears to mediate the behavioural efficacy of antidepressant medications [20]. Hippocampal neurogenesis is thought to be, at least partly, mediated by neurotrophins such as brain-derived neurotrophic factor (BDNF), levels of which are increased by antidepressant treatment in humans [21], and reduced by high fat/refined sugar (cafeteria) diets, as models of Western diets, in animal studies [22, 23].

In addition to affecting BDNF levels, animal research has shown that dietary components have a potent impact on hippocampal neurogenesis and function, and inflammatory processes. Foods such as omega-3 fatty acids, flavonoids, antioxidant-rich berries and resveratrol, a polyphenol found in red grapes and other fruits, stimulate neurogenesis, reduce oxidative activity and down-regulate pro-inflammatory processes [24, 25]. In contrast, high fat and high sugar foods reduce neuronal proliferation, contribute to an increased production of reactive oxygen species, and up-regulate pro-inflammatory processes, thus inducing increased neurodegeneration and impairments in learning and memory (for review, see [26]). However, as noted, the evidence demonstrating a detrimental impact of poor diet on the hippocampus is only available from animal studies to date. Thus, we aimed to examine the associations between dietary patterns and hippocampal volume in humans, taking into account other factors that may confound any detected relationships. We hypothesised that diets higher in nutrient and antioxidant-rich foods would be associated with larger hippocampal volumes, and that diets higher in saturated fats and refined carbohydrates would be associated with smaller hippocampal volumes. Using longitudinal data, we also investigated whether dietary patterns were associated with differential rates of atrophy over time.

## Methods

### Study design

Data were drawn from the Personality and Total Health (PATH) Through Life project, which is a longitudinal community study following three narrow age cohorts from Canberra and the neighbouring town of Queanbeyan in South-eastern Australia. The core aims of the PATH study are to delineate the course, risk and protective factors for depression, anxiety, substance use and cognitive ability across adulthood. A detailed description of the study is available elsewhere [27]. This analysis is focused on the oldest cohort, born between 1937 and 1941 and aged 60–64 years at the time of the baseline interview in 2001. This sample of participants was randomly drawn from the electoral roll (registration on the electoral roll is compulsory for Australian citizens) to produce a wave 1 sample of 2,551 (from a response rate of 58 %). Approximately 90 % of these participants who were still alive agreed to be re-interviewed 4 years later for the wave 2 assessment (n = 2,222). At each wave, participants took part in a structured interview (usually at their home or the Australian National University) that included a questionnaire completed using a hand-held computer, and physical and cognitive tests administered by a trained interviewer. Measures ranged from sociodemographic characteristics through to physical health, mental health, substance use, personality and cognition. Only measures used in the present study are described below.

This project draws on data from two PATH substudies. The magnetic resonance imaging (MRI) substudy has been detailed elsewhere [28, 29]. Of the 2,551 baseline participants, 2,076 agreed to be contacted for an MRI assessment, with 622 of these participants randomly selected and offered a scan. Of these, 431 also underwent an MRI scan at wave 2 and, after exclusion of those with an MRI abnormality, stroke or epilepsy, there were 341 respondents with MRI data from two waves.

At baseline, participants were also invited to take part in a *Diet and Health* substudy and given a self-completion questionnaire (including a food frequency questionnaire, detailed below), which was to be returned by mail when completed. Overall, 1,753 respondents (69 %) of PATH participants in the oldest cohort completed and returned the booklet. This analysis, therefore, is restricted to the 255 respondents who provided survey and MRI data at both waves, and completed and returned the food frequency questionnaire. A series of analyses compared the characteristics of this subsample to the larger sample of respondents not in this subsample but who had participated in the wave 2 PATH interview. The results showed no difference in terms of gender (*p* = .16), hypertension (p = .46), cognitive functioning (mini-mental state examination *p* = .14, spot-the-work *p* = .54), anxiety (*p* = .23) or depression symptoms (*p* = .22), life satisfaction (*p* = .99), reported diabetes (*p* = .39), or current smoking status (*p* = .18). Those included in the analytic sample were less likely to report heart problems (*p* = .04) or poor self-rated health (*p* = .02) and were more likely to report being married (*p* = .03) and to participate in regular moderate or vigorous exercise (*p* < .001).

After complete description of the study to the subjects, written informed consent was obtained from all participants prior to each wave of data collection in the PATH project. The study was approved by the Human Research Ethics Committee of The Australian National University.

### Measures

#### Sociodemographic and health covariates

Demographic covariates included age (in years) and gender. Educational attainment was operationalized by a binary variable indicating whether respondents had completed their high school certificate, and employment status differentiated between those who were working full-time or part-time, were unemployed and looking for work, or were no longer participating in the work force. Engagement in regular moderate or vigorous exercise was adapted from an approach used in the UK Whitehall II study [30], and the questionnaire assessed current smoking status. Health covariates included hypertension (respondents were classified as hypertensive if their systolic or diastolic blood pressure averaged over two readings were higher than 90 or 140 mmHg respectively, or if they reported use of antihypertensive medication), self-reported current diabetes, current depressive symptoms (Goldberg Depression Scale [31]) and reported use of antidepressant medication. Participants who reported a history of stroke or transient ischemic attack were excluded from the analyses.

#### Diet

Dietary intake was assessed using a version of the validated Commonwealth Scientific and Industrial Research Organisation Food Frequency Questionnaire (FFQ) [32]. The FFQ included a list of foods and standard serving sizes, and respondents indicated their habitual frequency of consumption on an 11-point scale (from never to three times a day) and indicated divergence from usual serving size. Dietary analysis of the data produced estimates of daily nutrient intake and (critical for this analysis) daily grams of each food item consumed. As detailed elsewhere [10], principal components analysis was used to summarize the information from the 188 distinct food items into meaningful scales representing dietary patterns. Two orthogonal factors labelled “prudent” (healthy) diet (characterized by the consumption of fresh vegetables, salad, fruit and grilled fish) and “Western” (unhealthy) diet (characterized by the consumption of roast meat, sausages, hamburgers, steak, chips, crisps and soft drinks) were identified. For each factor, higher scores represented greater levels of consumption, with a 1-point difference on each scale corresponding to one standard deviation (SD).

#### MRI images

All participants were imaged with a 1.5 T Philips Gyroscan ACS-NT scanner (Philips Medical Systems, Best, the Netherlands) for T1-weighted three-dimensional structural MRI in coronal orientation using a fast-field echo sequence. For wave 1, repetition time (TR) = 28.05, echo time (TE) = 2.64 ms, flip angle = 30°, matrix size = 256 × 256, field of view (FOV) = 260 × 260 mm, slice thickness = 2.0 mm and mid-slice to mid-slice distance = 1.0 mm, yielding over-contiguous coronal slices. For wave 2, TR = 8.93 ms, TE = 3.57 ms, flip angle = 8°, matrix size = 256 × 256 and FOV = 256 × 256 mm. Slices were contiguous with a slice thickness of 1.5 mm. Hippocampal and amygdalar volumes were determined by manually tracing the periphery of the region of interest (ROI) on each slice of a T1-weighted scan in coronal orientation using Analyze 5.0 (Brain Imaging Resource, Mayo Clinic, Rochester, MI, USA). The outlining of the hippocampus and amygdala always proceeded from anterior to posterior and was traced according to the protocol outlined by Watson et al. [33–35]. We repeated 16 volume estimations on 10 randomly selected scans, and interclass correlations between raters was in excess of 0.95 for all structures. Intracranial volume (ICV) was computed with the Freesurfer 5.3 package [36] for wave 1 and wave 2 images.

### Statistical analysis

After presenting descriptive data on baseline characteristics of the sample, the association between dietary factors and left and right hippocampal volumes over time was modelled using generalized estimating equation (GEE) models with a normal distribution, identity link function and exchangeable within-person working correlation structure. GEE models were used owing to the lack of independence of observations within respondents over the two time points (repeated measures). Hippocampal volume was normalized for ICV using the formula Vol_adj_ = vol – *b* × (ICV – mean ICV), where *b* is the regression coefficient of ROI volume on ICV. To correct for potential wave-specific procedural effects, the (centred) difference in overall ICV between wave 1 and wave 2 was also included as a covariate in these models [28]. Missing data for the items included in the current analysis was minimal (only four individuals reported missing data, with 2.1 % of data missing) and this was imputed by mean substitution or carrying forward (or backward) data. Additional models tested for an interaction between each of the dietary factors and wave on hippocampal volume to evaluate whether diet was associated with differential atrophy over time.

The robustness of the findings were evaluated through sensitivity analyses using a random intercept (rather than GEE) modelling approach, excluding respondents with missing data, inspection and exclusion of respondents with larger residual scores, and use of the log of hippocampal volume as the outcome measure. In all cases, results were consistent with those reported.

## Results

The baseline characteristics of the sample are presented in Table 1. Slightly less than half of the sample was female and the majority had completed their high school education. Just under half were working and, while most reported regular moderate or vigorous exercise, 62 % were identified as being overweight or obese. The mean time interval between the two MRIs was 4.0 years (SD = 0.21 years).

**Table 1:** Baseline characteristics

Characteristic	N (% or SD)
Female	118 (46 %)
Age (mean)	62.6 years (SD = 1.42)
Not completed high school	48 (17 %)
Physical activity	
None/mild	117 (46 %)
Moderate	106 (42 %)
Vigorous	32 (13 %)
Smoker	17 (7 %)
Body mass index	
Normal/under	96 (38 %)
Overweight	117 (46 %)
Obese	42 (16 %)
More than two depression symptoms	62 (22 %)
Reported antidepressant medications	18 (6 %)
Employed (full-time or part-time)	119 (41 %)

The results of longitudinal multivariable regression analyses in Table 2 show that both left and right hippocampal volume were significantly smaller at wave 2 than at baseline (declines of 327 mm^3^ and 236 mm^3^ respectively), after controlling for the range of covariates identified in the table (including age, gender, and potential differences and change in ICV). This difference, therefore, can be interpreted as the average age-related atrophy. The model also showed that being female was associated with significantly smaller left hippocampus volume, even after correcting for ICV.

**Table 2:** Results from random-intercept regression models, separate models for left and right hippocampal volume

Covariates	Left hippocampal volume		Right hippocampal volume	
	β (SE)	*p* value	β (SE)	*p* value
Prudent (healthy) diet	48.7 (22.8)	.032	17.6 (22.9)	.442
Western (unhealthy) diet	−52.6 (26.9)	.05	−30.6 (27.0)	.256
Wave	−327.5 (26.2)	< .001	−235.8 (27.4)	< .001
Sex (ref = male)	−289.3 (46.5)	< .001	−294.4 (46.7)	< .001
Baseline age (years)	1.77 (14.5)	.903	6.9 (14.6)	.635
Depression symptoms	−6.0 (9.9)	.546	2.8 (10.1)	.780
Reported depression medication	−78.2 (90.3)	.386	−146.2 (90.8)	.107
Working (ref = not working)	47.1 (36.0)	.191	75.1 (36.9)	.042
Did not complete high school	−34.5 (58.3)	.554	−82.0 (58.7)	.162
Regular exercise				
Moderate	−37.5 (44.3)	.397	−12.2 (44.5)	.784
Vigorous	106.8 (67.0)	.111	32.7 (67.4)	.628
Current smoker	90.7 (84.1)	.280	79.3 (84.5)	.348
Diabetes	−160.8 (86.6)	.063	−66.3 (87.1)	.446
Hypertension	−39.1 (43.8)	.372	−13.5 (44.0)	.758
Constant	3066.9		2735.5	

Hippocampal volume was corrected for intracranial volume. Other covariates included elapsed time between magnetic resonance imaging and change in intracranial volume over time. *SE* standard error

**Fig. 1:**
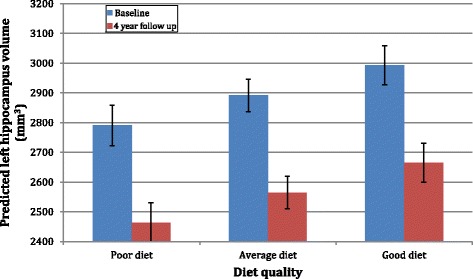
Predicted left hippocampal volume (with standard errors represented by error bars) at baseline and 4-year follow-up for respondents classified with poor, average and good quality diet based on scores on the Western and prudent dietary factor scores (*poor* defined as 1 SD below mean on prudent and 1 SD above mean on Western dietary factor scores; *average* defined as mean/0 on both prudent and Western dietary factor scores; *good* defined as 1 SD above mean on prudent and 1 SD below mean on Western dietary factor scores)

Salient to our hypotheses, both dietary factors were associated with left hippocampal volume. Every SD increase in healthy ‘prudent’ dietary pattern was associated with a 45.7 mm^3^ (SE 22.9) larger left hippocampal volume, while higher consumption of an unhealthy ‘western’ dietary pattern was associated with a 52.6 mm^3^ (standard error [SE] 26.6 mm^3^) smaller left hippocampal volume. These two effects were independent of each other and over and above the effect of other covariates. There was no evidence of an interaction between the dietary factor scores (i.e. the effects were additive not multiplicative; β = −30.0, SE = 26.2, *p* = 0.25), or between each of the dietary factor scores and time (i.e. diet was not associated with differential atrophy: β_prudent × time_ = 20.8, SE = 24.4, *p* = 0.40; β_western × time_ = 27.2, SE = 28.8, *p* = 0.34). To better illustrate the overall association between diet and left hippocampus volume, we modelled predicted hippocampal volume for three categories of respondents: those with a healthy diet (defined as 1 SD above mean on prudent and 1 SD below mean on Western dietary factor scores); those with an average diet (mean/0 on both prudent and Western dietary factor scores); and those with an unhealthy diet (1 SD below mean on prudent and 1 SD above mean on Western dietary factor scores). All other covariates were held constant at the mean or most common value. The predicted baseline and wave 2 left hippocampal volume for these three groups is presented in Fig. 1. These estimates were derived from the final statistical model and were not based on measures from specific respondents. Overall, the data suggest that 6.6 % and 8.2 % of respondents would have scores on a combined Western and prudent dietary factor that would have them classified with good or poor diets respectively.

The difference in left hippocampal volume between poor and average, and between average and good diets was statistically significant (β = 101.3, SE = 38.2, *p* = 0.008). Thus, the figure shows that the difference in hippocampal volume between those classified with a healthy and or unhealthy diet was 203 mm^3^ (95 % confidence interval, 54–352 mm^3^), a difference which corresponds to 62 % of the average decline in left hippocampal volume observed over the 4-year period.

The results from the second GEE model provided no evidence of any association between the dietary factors and right hippocampal volume; however, it is worth noting that the coefficients associated with the prudent and Western dietary factors were in the direction consistent with the left hippocampus results. Again, the right hippocampus analysis provided no evidence of an interaction between the two dietary factor scores (β = −41.9, SE = 26.3, *p* = 0.11), or between either of the dietary factor scores and time (β_prudent × time_ = 12.8, SE = 25.7, *p* = 0.62; β_western × time_ = −9.4, SE = 30.2, *p* = 0.76).

## Discussion

In this study, both lower scores on a healthy, nutrient-dense dietary pattern and higher scores on an unhealthy dietary pattern were independently associated with smaller left hippocampal volumes in older adults. These findings are concordant with extensive data from animal studies indicating that aspects of diet have an impact on neurotrophins, neurogenesis and hippocampal function. Although we observed significant decline in both left and right hippocampal volume over the 4-year period of the study, there was no evidence that dietary pattern was associated with differential rates of change in hippocampal size over time. Rather, poor quality diet (greater consumption of an unhealthy diet, lower consumption of a healthy diet) and ageing (the time between assessments) were both independently associated with smaller left hippocampal volumes.

In animal studies, a high-fat and sucrose (HFS) diet, as a model of a Western diet, reduces levels of BDNF, leading to impairments in neuronal plasticity, learning and behaviour [22]. The long-term intake of such a diet produces cognitive deficits, especially in tasks that require the hippocampus [37]. Importantly, duration of exposure to an HFS diet is related to the extent of the decrease in BDNF and the degree of learning and memory impairment in animal models, suggesting that the longer the exposure to these sorts of dietary components, the more profound the impact on hippocampal functioning and brain plasticity [22]. However, even short-term feeding of the HFS diet results in cognitive impairment and potentiates the negative impact of mild brain injury on BDNF levels, synaptic plasticity and cognitive functioning [38]. A recent study showed that a diet rich in sugar and fat or rich in sugar alone resulted in impairments in hippocampal-dependent memory, independently of weight changes, in as little as five days. Interestingly, the sucrose-only diet was associated with increases in hippocampal inflammation and oxidative stress that were not observed in the rats fed a high fat and sugar diet combined [39]. There is evidence to suggest that the impact on hippocampal-dependent memory of a high fructose diet may relate to increases in plasma triglycerides [40]. In another study utilizing a Western diet (40 % butterfat and 29 % sucrose) or a very high fat lard diet (60 % fat) in aged rodents, only the very high fat lard diet increased hippocampal oxidative damage and impaired retention in behavioural tests [41].

Some hippocampal-associated cognitive impairments appear reversible by supplementation with vitamin E [42] or omega-3 fatty acids [43] in animal studies. Certainly there are data indicating a beneficial effect of ‘healthy’ dietary components on neurogenesis and the hippocampus. There is evidence that dietary polyphenols, such as flavonoids, increase BDNF and neurogenesis in animal models [44–46], as do diets rich in polyphenols and polyunsaturated fatty acids combined [47]. Long-chain omega-3 fatty acids alone also promote neurogenesis in vitro and in vivo [48]. However, the possible application of results from such supplement studies should be considered with caution; consistently, the use of dietary supplements in humans does not result in health benefits equivalent to dietary sources of nutrition (e.g. [49]) and the focus should continue to be on overall dietary patterns and quality.

There are limited data on diet and hippocampal-related parameters in humans; however, adults with schizophrenia who were undertaking a regime of dietary improvement for at least 4 weeks had higher serum levels of BDNF than those in the no diet condition [50]. Sedentary but otherwise healthy males who ate a high fat diet for 1 week performed worse on tasks measuring attention and speed of retrieval than they had prior to the diet [51]. Less directly, healthy diets are often, although not always [52], associated with better cognitive functioning [53], and a reduced risk of dementia [54] and cognitive decline [7], while unhealthy diets are associated with increased cognitive impairment [55]. Null findings in such studies [52] may be due to interactions with other risk factors or an artefact of the dietary scoring method used to define a healthy diet; for example, use of Mediterranean dietary indices may not necessarily be optimal outside of regionally specific contexts.

It is notable that diet quality was only significantly associated with the left and not the right hippocampus. A number of previous investigations have suggested that the left hemisphere and particularly the left hippocampus may be more prone to neurodegeneration. For example, it has been shown in Alzheimer’s disease that cortical atrophy occurs earlier and progresses faster in the left hemisphere [56]; recent meta-analyses showed that the left hippocampus is smaller than the right in Alzheimer’s disease and mild cognitive impairment [57] but not in normal ageing [58]; and Giannakopoulos and colleagues [59] demonstrated that micro-vasculature pathology was more prevalent in the left than the right hemisphere. Consequently, it is possible that the laterality effect observed in the present study reflects a greater vulnerability of the left hippocampus to the adverse effects of poor diet. Alternatively, random measurement noise and population variability may also have contributed to this effect.

### Strengths and limitation

The strengths of our study include the use of data drawn from a large, longitudinal population-based sample of adults, with well-validated tools used for the assessment of both diet and depression. Outcomes of the diagnostic measures used for depression in PATH are concordant with national prevalence rates for depression and with established age and gender differences [27]. Although we observed a statistically significant decline in hippocampal volume over the 4-year follow-up period, and showed that poorer diet quality was associated with smaller left hippocampal volume, we found no evidence of different rates of hippocampal atrophy over the follow-up period for those reporting different dietary patterns. This may reflect the relatively small sample size, the relative size of the dietary effects (i.e. compared to general age-related atrophy or measurement differences), or the relative narrowness of the follow-up period, which all may have limited our capacity to observe differential relationships between dietary exposures and hippocampal atrophy over the follow-up period. Over a longer follow-up period, it may be possible to separate the risk or protective dietary effects from the general age-related atrophy. Alternatively, it may be that the narrow age range studied here does not correspond to the point in the life course at which diet-related atrophy is strongest. It may be, for example, that the adverse effects of an unhealthy diet on the brain occur prior to age 60, or that the protective effects of a healthy diet are more evident in hippocampal volume at older ages and offset the general rate of atrophy.

Additionally, while we corrected our analyses for differences in ICV between waves 1 and 2, these corrections may only have partly controlled for differences in scanning protocols between waves. However, this is unlikely to have important implications for the findings presented because no significant effect was detected in relation to longitudinal change. On the other hand, these protocol differences may partly explain the null findings in our longitudinal analyses. Finally, we assumed stability of dietary intakes over the 4-year interval; however, there may have been some variability that we were unable to monitor.

## Conclusion

In this cohort study of community-based older adults, lower intakes of nutrient-dense foods and higher intakes of unhealthy foods were each independently associated with smaller left hippocampal volumes. To our knowledge, this is the first human study to demonstrate associations between diet and hippocampal volume concordant with data previously observed in animal studies. These findings suggest the potential for dietary interventions to promote hippocampal health, decrease age-related atrophy, and prevent negative health outcomes associated with hippocampal atrophy. They also support the extensive data from human observational and intervention studies showing that unhealthy dietary patterns are associated with increased prevalence or risk, and healthy dietary patterns with reduced risk, of depression [6, 7, 60] and reinforce the imperative to improve dietary intakes at the population level and in clinical settings for better mental health outcomes [5].
